# 268. Methicillin Sensitive Versus Methicillin Resistant *Staphylococcus aureus* Nosocomial Meningitis

**DOI:** 10.1093/ofid/ofab466.470

**Published:** 2021-12-04

**Authors:** Deniz Akyol, Selin Bardak özcem, Sinan Mermer, Şöhret Aydemir, Tansu Yamazhan, Bilgin Arda, Erkin Özgiray, Taşkın Yurtseven, Sercan Ulusoy, Oğuz Reşat Sipahi

**Affiliations:** 1 Doctor, İzmir, Izmir, Turkey; 2 Ege University Faculty of Medicine, İzmir, Izmir, Turkey; 3 Professor Doctor, Izmir, Izmir, Turkey

## Abstract

**Background:**

Herein, we aimed to analyze the outcomes of the methicillin sensitive (MS) versus methicillin resistant (MR) culture-proven Staphylococcus spp. nosocomial meningitis (S-NM) in our setting.

**Methods:**

We extracted data and outcomes for all adult patients (age >18 years) consulted by the Infectious Diseases Consultants and diagnosed NM (developed at a compatible time according to CDC nosocomial meningitis definitions) between January 2006 and 2021 and fulfilled the following study inclusion criteria: (a) Age ≥18-year-old; (b) CSF culture is positive for *Staphylococcus* spp. (c) Presence of at least two of three clinical/laboratory criteria as meningitis findings: (i) Body temperature >38^o^C; (ii) CSF finding; >250 leucocytes/mm^3^; (iii) at least one of the following clinical findings, ie. impairment of consciousness, neck stiffness, nausea/vomiting. Identification of the infecting bacteria and determination of antimicrobial susceptibility were performed using the VITEK 2 automated system (BioMerieux Inc, Mercy L’etoil, France) and conventional methods. Resistance to methicillin was tested by E-test (bioMérieux). Antibacterial susceptibility tests were evaluated according to Clinical Laboratory Standards Institute (CLSI) criteria until 2014 and EUCAST between 2015 and 2021. Chi-square and Student T tests were used for statistical comparison.

**Results:**

A total of 9 patients in MSS-NM, 41 patients in MRS-NM group fulfilled the study inclusion criteria. Age, gender, and CSF findings (except CSF glucose was significantly lower in MSS-NM) were similar in both groups (Table 1). Besides, EOT clinical success and overall success (EOT success followed by one-month survival without relapse or reinfection) rates were similar (Table 1). Relapse and reinfection rates during post-treatment one month period were 0%-0% and 0%-6.6% in MSS/MRS-NM, respectively. In MRS-NM group reinfection pathogens were *Acinetobacter baumannii* and *Pseudomonas aeruginosa* after 12 and 30 days end of treatment.

Characteristics of NM

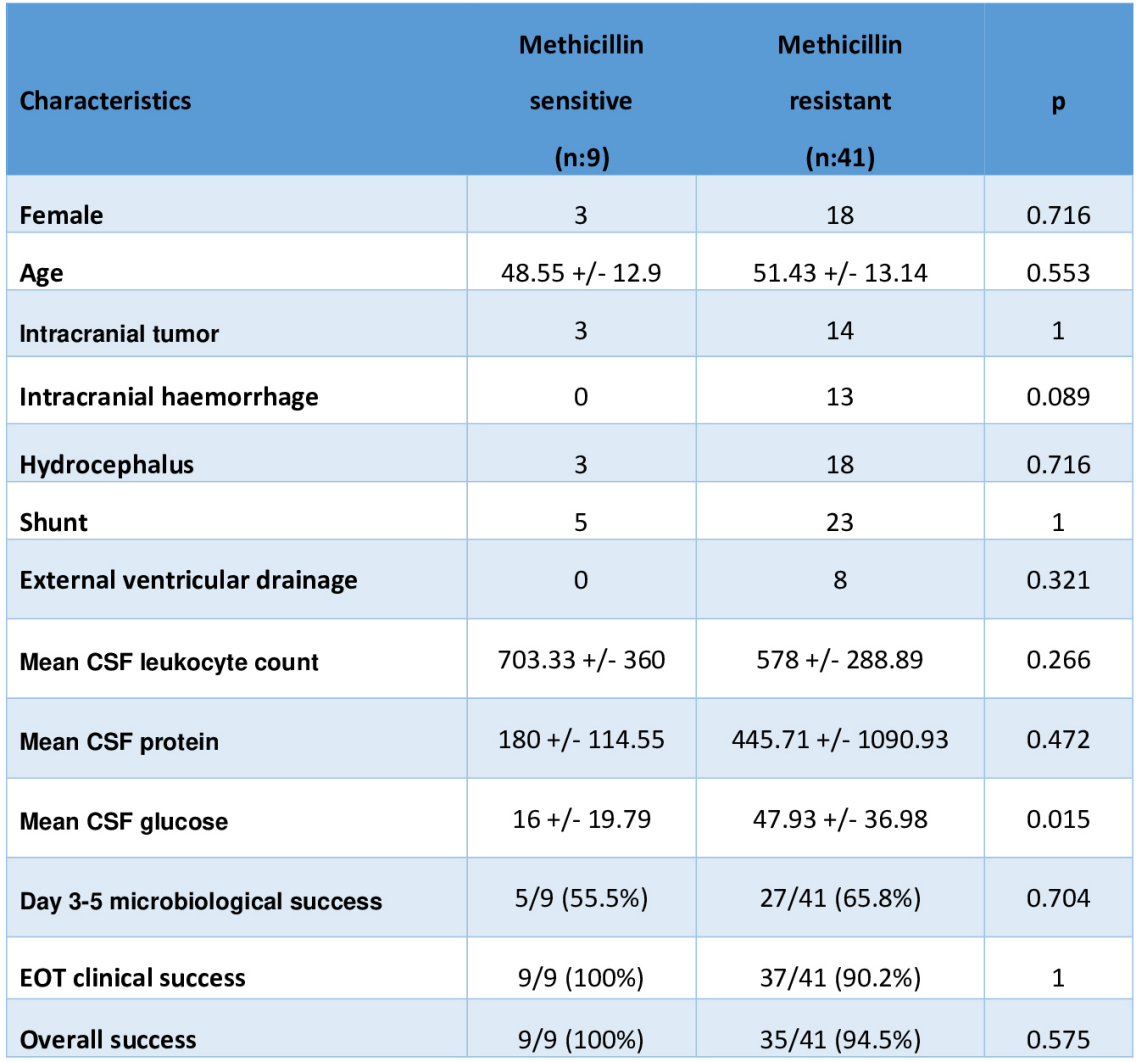

**Conclusion:**

Overall success in MSS-NM was acceptable while it was non-significantly lower in MRS-NM. The medical community should seek better infection control measures from NM.

**Disclosures:**

**All Authors**: No reported disclosures

